# Adaptive responses of *Chlamydomonas reinhardtii* to cadmium stress: physiological, biochemical, and lipidomic insights

**DOI:** 10.1016/j.btre.2026.e00970

**Published:** 2026-07-08

**Authors:** Mohammad Aadil, Nihad Sahri, Mutale Joan Chanda, Soufiane Fal, Rachid Benhida, Hicham El Arroussi, Mohammed Danouche

**Affiliations:** a College of Chemical Sciences and Engineering (CCSE), Department of Chemical and Biochemical Sciences - Green Process Engineering (CBS-GPE), Mohammed VI Polytechnic University (UM6P), Ben Guerir 43150, Morocco; b Physio-Chemical Laboratory of Inorganic and Organic Materials (LPCMIO), Materials Science Center (MSC), Ecole Normale Supérieure, Mohammed V University in Rabat, 10100, Rabat, Morocco; c Algal Biotechnology laboratory, Agrobiosciences, college of Agriculture and Environmental Sciences (CAES), Mohammed VI Polytechnic University (UM6P), Ben Guerir, Morocco; d Institute of Chemistry, Nice UMR 7272, Côte d’Azur University, French National Center for Scientific Research (CNRS), Nice, France

**Keywords:** Cadmium, Chlamydomonas reinhardtii, Fatty acid metabolism, Oxidative stress, Lipidomics

## Abstract

Cadmium (Cd), a toxic heavy metal, poses a significant threat to aquatic ecosystems by impairing primary producers such as microalgae. This study investigated the adaptive responses of *Chlamydomonas reinhardtii* to Cd stress through physiological, biochemical, and GC-MS-based lipidomic analysis. Cultures were exposed to the EC_50_ concentration of Cd (20 ppm) for 8 days. Cd exposure significantly increased oxidative stress markers, including hydrogen peroxide (+69%), malondialdehyde (+336%), and proline (+144%). Total carbohydrate and lipid contents increased by 21.7% and 14%, respectively, whereas protein and total photosynthetic pigment contents decreased by 39.5% and 44%, respectively, indicating metabolic reprogramming under Cd stress. Antioxidant capacity was enhanced as reflected by a 50% reduction in the DPPH IC_50_ value. Lipidomic analysis revealed a decrease in unsaturated fatty acids accompanied by the accumulation of lipid-derived aldehydes and saturated hydrocarbons. KEGG pathway enrichment analysis identified significant alterations in fatty acid biosynthesis, fatty acid elongation, glutathione metabolism, and butanoate metabolism, highlighting coordinated oxidative and lipid metabolic reprogramming as key adaptive responses to Cd stress.

## Highlights

• Cadmium exposure induces strong oxidative stress in *Chlamydomonas reinhardtii.*
• Cd stress triggers carbon reallocation from proteins toward sugars and lipids.
• Lipidomics reveals a shift from PUFAs to saturated and long-chain fatty acids.
• Oxidized lipids (*e.g.,* 2-nonenal) emerge as biomarkers of Cd-induced stress.
• Integrated lipid–redox remodeling underpins Cd tolerance in microalgae.

## 1 Introduction

Heavy metals (HMs) are naturally occurring elements in the lithosphere and generally remain harmless when confined to their geological compartments, where they exist in equilibrium through natural processes such as leaching, complexation, and precipitation. However, environmental contamination occurs when these elements are mobilized by anthropogenic activities, including mining, industrial processes, and improper waste management [1,2]. Once mobilized, HMs can exert toxic effects by disrupting cellular homeostasis and inducing oxidative stress in exposed organisms. Among them, cadmium (Cd) is considered one of the most hazardous environmental pollutants because of its high toxicity, mobility and persistence. Cd readily enters aquatic ecosystems, where it accumulates in water bodies and sediments and is efficiently taken up by primary producers, particularly phytoplankton and aquatic microalgae [3]. Unlike essential trace metals, Cd has no known biological function and exerts toxicity by displacing essential metal cofactors, inhibiting enzyme activity, compromising membrane integrity, and disrupting photosynthetic and respiratory processes [4,5].

Microalgae are highly sensitive to HMs contamination and are widely used as model organisms to investigate the cellular and metabolic consequences of HMs toxicity. At the cellular level, Cd disrupts redox homeostasis, triggering excessive production of reactive oxygen species (ROS), including singlet oxygen (^1^O_2_), superoxide anion (O_2_^•−^), hydroxyl radical (^•^OH), perhydroxyl radical (HO_2_^•^), and hydrogen peroxide (H_2_O_2_). The accumulation of these reactive species induces lipid peroxidation, protein oxidation, carbohydrate degradation, and DNA damage, ultimately impairing growth, photosynthetic efficiency, and metabolic performance [6,7].

To mitigate oxidative damage, microalgae activate a range of adaptive responses involving non-enzymatic antioxidants, including glutathione, proline, ascorbate, and carotenoids, as well as enzymatic defenses such as superoxide dismutase, catalase, ascorbate peroxidase, and glutathione peroxidase [8]. Beyond antioxidant defense, Cd exposure also induces osmotic adjustment and metabolic reprogramming, altering the synthesis and turnover of central metabolites, including lipids [9]. Such metabolic plasticity is a key component of microalgal stress tolerance; however, its underlying biochemical and lipidomic mechanisms under Cd stress remain incompletely understood. In this context, *Chlamydomonas reinhardtii*, a unicellular green alga, is a well-established model organism for studying photosynthesis, redox regulation, and environmental stress responses. Its fully sequenced nuclear, mitochondrial, and chloroplast genomes, together with its genetic tractability and ease of cultivation, make it an excellent system for investigating cellular stress responses and metabolic regulation [10]. Although Cd toxicity in *C. reinhardtii* has been investigated at the physiological and biochemical levels, existing studies have primarily focused on enzymatic antioxidant responses, metal accumulation, and changes in gene expression [11,12], leaving the lipidomic aspects of the stress response poorly characterized. In particular, how Cd stress reshapes the cellular lipid landscape, including fatty acid saturation, alkane composition, sterol content, and lipid-derived oxidation products, and how these alterations are mechanistically linked to photosynthetic impairment and carbon reallocation remains largely unresolved. Under Cd exposure, alterations in membrane lipid composition, fatty acid saturation, and the accumulation of lipid-derived oxidation products contribute to the maintenance of membrane integrity and cellular homeostasis during oxidative stress [22]. However, comprehensive characterization of these lipidomic changes remains limited in *C. reinhardtii*. To address this knowledge gap, gas chromatograph coupled to a mass spectrometer (GC-MS) based lipidomic profiling represents a suitable analytical approach for the characterization of fatty acid methyl esters (FAMEs), hydrocarbons, and lipid-derived aldehydes associated with oxidative lipid remodeling. Compared with LC-MS, which is primarily designed for the analysis of intact polar lipids, GC-MS provides high chromatographic resolution and reliable library-based identification of volatile and derivatized lipophilic metabolites [23]. Therefore, this present study aimed to characterize the responses of *C. reinhardtii* to Cd stress by integrating physiological and biochemical analyses, with GC-MS-based lipidomic profiling and KEGG pathway enrichment analysis. This approach provides new insights into membrane lipid remodeling and the associated metabolic alterations underlying the cellular stress response to Cd stress.

## 2 Materials and methods

### 2.1 Microalgal strain, cultivation conditions, and Cd stress application

*C. reinhardtii* was obtained from the microalgae collection of the Algal Biotechnology Laboratory (Benguerir, Morocco) and cultivated in BG_11_ medium as previously described by Danouche et al. [8]. Pre-cultures were progressively scaled up from 6-well plates to 50, 250, and 500 mL Erlenmeyer flasks to obtain physiologically stable inocula. Cultures were maintained under a 10 h light/14 h dark photoperiod at 25 ± 2 °C with orbital shaking at 150 rpm. Cd stress was induced using CdCl_2_ (99.0% purity, Alfa Aesar). A sterile stock solution (1 g L⁻^1^) was prepared in distilled water and sterilized by filtration (0.22 µm membrane filter). Working concentrations (0, 5, 10, 15, 20, 25, 35, 50, 75, 100, 150, and 200 ppm) were prepared by dilution in BG_11_ medium. Cultures were inoculated at an initial OD_680nm_ of 0.500 ± 0.025 and dispensed into 12-well CellBIND® microplates (3 mL per well, n = 4). Growth was monitored spectrophotometrically at OD_680nm_ (Ultrospec™ 3100 Pro, Amersham Biosciences), and the EC_50_ value was determined after 8 days of exposure. EC_50_ values are expected to vary between small-scale (microplate) and larger-scale culture systems because of differences in physicochemical conditions. In microplate cultures, reduced buffering capacity, limited gas exchange, and heterogeneous light distribution may increase the apparent sensitivity of cells to Cd stress [13]. In contrast, the principal experiments were conducted in 2 L flasks under continuous aeration with pressurized air bubbling, which provided improved gas exchange, more homogeneous light distribution, and enhanced mixing. These conditions promoted greater biomass buffering capacity and a more stable physiological environment, thereby moderating the cellular response to Cd stress [14]. Therefore, the Cd concentrations used for subsequent physiological, biochemical, and lipidomic analyses were selected with consideration of this scale-dependent difference in stress response.

### 2.2 Determination of microalgal growthand biomass productivity

Biomass production was evaluated under two experimental conditions: a control group cultured in Cd-free BG_11_ medium and a treatment group cultured in BG_11_ medium supplemented with Cd at the previously determined EC_50_ concentration (20 ppm). The specific growth rate (µ) was calculated using Eq. (1). Where N_1_ and N_2_ represent the OD_680nm_ measured at times T_1_ and T_2_, respectively.

(1) $$\mu \;=\;\frac{\text{ln}\left(N2/N1\right)\;}{\left(T2\;-\;T1\right)\;}$$

For dry weight determination, a known volume of culture was filtered, and the retained biomass was dried in an oven at 50 °C for 48 h before being weighed. Biomass productivity was calculated according to Eq (2).

(2) $$\text{Biomass}\;\text{productivity}\;\left(gL^{-1}d^{-1}\right)=\frac{\text{Biomass}\;\text{yield}\;\left(gL^{-1}\right)}{\text{Number}\;\text{of}\;\text{days}\;}$$

### 2.3 Determination of oxidative stress biomarkers and metabolic responses

To evaluate the biochemical responses of *C. reinhardtii* to Cd stress, biomarkers and the key metabolic parameters were analyzed. After 8 days of cultivation, cells from control and Cd-exposed cultures were harvested by centrifugation and used for subsequent biochemical analyses.

#### 2.3.1 Determination of hydrogen peroxide content

H_2_O_2,_ a key ROS, was used as an indicator of oxidative stress. Its content was quantified according to the method of Velikova et al. [15], with minor modifications. Fresh biomass (0.5 g) was homogenized in 5 mL of ice-cold 1% (w/v) trichloroacetic acid (TCA) and centrifuged at 12,000 × g for 10 min at 4 °C. An aliquot (0.5 mL) of the supernatant was mixed with 1.5 mL of 50 mM potassium phosphate buffer (pH 7.0) and 1 mL of 1 M potassium iodide. The reaction mixture was incubated in the dark for 1 h, and absorbance was measured at 390 nm. The H_2_O_2_ concentration was calculated from a standard calibration curve.

#### 2.3.2 Determination of lipid peroxidation

Lipid peroxidation was assessed by measuring malondialdehyde (MDA) content using the thiobarbituric acid reactive substances (TBARS) assay according to the method of Esterbauer and Cheeseman [16]. Fresh biomass (0.5 g) was homogenized in 5 mL of 1% (w/v) TCA using an ultrasonic homogenizer on ice and centrifuged at 6300 × g for 10 min at 10 °C. An aliquot (0.5 mL) of the supernatant was mixed with 2 mL of 20% (w/v) TCA containing 0.5% (w/v) thiobarbituric acid (TBA). The reaction mixture was heated at 96 °C for 25 min, rapidly cooled on ice, and centrifuged at 10,000 × g for 10 min. The absorbance was measured at 532 nm, and MDA concentration was calculated using an extinction coefficient of 159.2 mM^−1^ cm^−1^.

#### 2.3.3 Determination of proline content

Proline, an important osmoprotectant and stress-related metabolite, was quantified according to the method of Bates et al. [17]. Lyophilized biomass (50 mg) was homogenized in 3 mL of 3% (w/v) sulfosalicylic acid and centrifuged at 4000 × g for 15 min. An aliquot (2 mL) of the supernatant was mixed with 2 mL of glacial acetic acid and 2 mL of acid ninhydrin reagent, followed by incubation in a boiling water bath for 45 min. After cooling, 4 mL of toluene was added to extract the chromophore, and the absorbance was measured at 520 nm. Proline concentration was determined from an l-proline standard curve and expressed as µg g⁻^1^ DW.

#### 2.3.4 Determination of radical scavenging activity

Radical scavenging activity was evaluated using the 2,2-diphenyl-1-picrylhydrazyl (DPPH) assay according to the method of Jayshree et al. [18]. Methanolic extracts were prepared from lyophilized biomass of control and Cd-exposed *C. reinhardtii* cultures and tested at concentrations ranging from 1.25 to 25 mg mL^−1^. An aliquot (20 µL) of each extract was mixed with 180 µL of DPPH solution (60 µM in methanol) in 96-well microplates and incubated in the dark at room temperature for 30 min. The absorbance was measured at 517 nm. Radical scavenging activity was expressed as percentage inhibition of DPPH radicals, and the concentration required to inhibit 50% of the DPPH radicals (IC_50_) was determined

#### 2.3.5 Determination of the biochemical composition

To evaluate metabolic responses of *C. reinhardtii* to Cd stress, its biochemical composition was determined. Photosynthetic pigments were extracted from control and Cd-exposed cells at a biomass concentration of 1 mg mL^−1^ using 95% (v/v) ethanol, following Danouche et al. [8]. The absorbance spectra of pigment extracts were first recorded over the wavelength range of 200–800 nm using a UV–Vis spectrophotometer (V-770 UV–Visible/NIR, JASCO) to assess pigment composition and identify potential Cd-induced spectral changes. Pigment concentrations were subsequently calculated using Eqs. (3–5).

(3) $$\begin{array}{cccc} & \text{Chlorophyll}\;a=13.36\times A_{664}-5.19\times A_{649} & & \end{array}$$

(4) $$\begin{array}{cccc} & \text{Chlorophyll}\;b=27.43\times A_{649}-8.12\times A_{664} & & \end{array}$$

(5) $$\begin{array}{cccc} & \text{Total}\;\text{carotenoids}=\frac{1000\times A_{470}-2.13\times \text{Chlorophyll}\;a-97.63\times \text{Chlorophyll}\;b}{209} & & \end{array}$$

Total soluble sugars were quantified using the phenol-sulfuric acid method according to Dubois et al. [19], with glucose used as the calibration standard and absorbance measured at 490 nm. Proteins were extracted by combination of heat treatment and sonication and subsequently quantified using the Bradford assay [20], with bovine serum albumin as the standard.

### 2.4 GC-MS based lipidomic analysis

#### 2.4.1 Lipid extraction

Total lipids were extracted and quantified according to the method of Bligh and Dyer [21], with slight modifications. Briefly, dried biomass was homogenized in a solvent mixture of chloroform, methanol and water (2:1:1, v/v/v). The homogenate was centrifuged at 4700 rpm for 5 min, and lower chloroform phase containing lipids was collected and evaporated to dryness under a gentle nitrogen stream. The resulting lipid residue was weighed gravimetrically.

#### 2.4.2 Lipids transesterification and GC-MS analysis

Extracted lipids were derivatized into FAMEs by acid-catalyzed transesterification. Briefly, 2 mL of methanol containing 6% (v/v) sulfuric acid was added to each lipid extract, followed by incubation at 90 °C for 2 h. The reaction mixture was then ultrasonicated at 40 kHz for 1 h to improve transesterification efficiency. After solvent evaporation, phase separation was achieved by adding chloroform and distilled water (2:1, v/v). Dodecane was added as an internal standard for peak area normalization [24]. GC-MS analyses were performed using an Agilent 7890A GC-MS equipped with an HP-5MS capillary column (30 m × 0.25 mm × 0.25 µm). Helium was used as the carrier gas at a constant flow rate of 1.5 mL min⁻^1^, and sample were injected in split mode (1:4). The oven temperature was programmed from 30 to 270 °C using successive heating ramps of 10, 30, and 5 °C min⁻^1^. The ion source and quadrupole temperatures were maintained at 230 and 150 °C, respectively. Mass spectra were acquired in full-scan mode over an *m/z* range of 30 to 1000, and compounds were identified by comparison with the NIST 2017 mass spectral library [25].

#### 2.4.3 Lipidomic data processing

GC-MS data from control and Cd- exposed cultures were processed using an internal standard-based approach, with peak areas normalized to dodecane to obtain relative abundance of lipid metabolites [26]. Following compound identification, lipid features were curated based on analytical reliability and biological relevance, and only lipid species previously reported in *C. reinhardtii* or closely related microalgal species were retained for subsequent analysis (Table S1). The identified metabolites were classified into chemical categories, and the relative abundances of each lipid class was calculated under control and stressed conditions (Table 1, Table 2, Table 3). The dataset was exported as a .csv file and analyzed using MetaboAnalyst 5.0. Prior to statistical analysis, data were log_10_-transformed and mean-centered to improve normality and reduce systematic variation (Figs. S2 and S3). Multivariate analyses, including feature-impact biplots, and hierarchical clustering using Ward’s linkage and Euclidean distance, were performed to evaluate stress-induced lipidomic shifts. Functional analysis was conducted using KEGG-based metabolite set enrichment and correlation patterns analyses were performed and visualized using Python (Pandas, NumPy, Matplotlib).

**Table 1:** Distribution of FAMEs in *C. reinhardtii* under control and Cd-stressed conditions.

Class	Compounds	Control (µg mg^−1^)	Cd-stressed (µg mg^−1^)	Relative change (%)
SFA	C14:0	n/d	21.5	+100
	C16:0	312.8	579.0	+85.1
	C16:0 (branched)	8.1	n/d	−100
	C18:0	103.4	251.6	+143.3
	C18:0 (branched)	88.8	n/d	−100
	C22:0	3.0	26.5	+786.7
	C25:0	n/d	69.3	+100
	C28:0	n/d	89.3	+100
**Total SFA**	516.1	1037.4	+100.9	
MUFA	C16:1	n/d	5.9	+100
	C18:1 (elaidic)	n/d	7.1	+100
	C18:1 (oleic)	n/d	2.6	+100
	C18:1 (petroselinic)	n/d	7.3	+100
	C19:1	n/d	2.8	+100
**Total MUFA**	0	25.7	+100	
PUFA	C16:3 (isomer 1)	3.6	n/d	−100
	C16:3 (isomer 2)	16.5	n/d	−100
	C18:4	2.2	n/d	−100
	C20:4 (arachidonic)	13.3	n/d	−100
**Total PUFA**	35.7	0	−100

**Table 2:** Distribution alkanes and alkenes in *C. reinhardtii* under control and Cd-stressed conditions.

Compounds	Control (µg mg^−1^)	Cd-stressed (µg mg^−1^)	Relative change (%)
1-Eicosene	7.7	n/d	−100
1-Octadecene	8.7	5.6	−35.3
1-Docosene	n/d	5.8	+100
1-Nonadecene	1.8	n/d	−100
1-Hexacosene	n/d	0.9	+100
**Alkene Total**	18.2	12.3	−32.4
3,8-Dimethyldecane	n/d	7.7	+100
Tetratriacontane	11.5	7.8	−32.1
1-Bromoeicosane	n/d	8.6	+100
Tetratetracontane	2.7	9.3	+239.9
Heneicosane, 11-decyl-	n/d	10.0	+100
Hexadecane, 1-iodo-	n/d	13.7	+100
2-Methyltriacontane	5.4	2.0	−63.3
Octadecane, 1-chloro-	n/d	2.9	+100
Tritetracontane	0.2	3.0	+1086.5
Docosane, 1,22-dibromo-	0.9	3.4	+260.4
7-Hexyltridecane	n/d	3.8	+100
3-Methyltritriacontane	n/d	5.0	+100
Octadecane, 1-iodo-	15.6	5.5	−65.0
Octacosane, 2-methyl-	4.9	n/d	−100
Tetracosane	0.6	n/d	−100
Tricosane	15.8	n/d	−100
Pentatriacontane	n/d	0.2	+100
Heneicosane, 11-pentyl-	n/d	0.3	+100
Docosane	10.0	0.4	−96.1
Tetracontane	n/d	0.7	+100
1-Bromodocosane	2.2	1.0	−55.6
Nonadecane	n/d	1.3	+100
1,1′-[(1-Methyl-1,2-ethanediyl)bis(oxy)]bis-octadecane	n/d	1.6	100
Hexacosane, 1,26-dibromo-	4.8	n/d	−100
Dotriacontane, 1,32-dibromo-	6.0	n/d	−100
Chloroicosane	2.0	n/d	−100
3-Methylheneicosane	3.0	n/d	−100
2-Methylhentriacontane	14.0	n/d	−100
2,6,10-Trimethyltridecane	1.7	n/d	−100
1-Chlorodocosane	2.1	n/d	−100
1-Bromo-4-bromomethyldecane	1.3	n/d	−100
1-Bromotetracosane	n/d	2.3	+100
17,21-Dimethylheptatriacontane	8.9	n/d	−100
1,14-Dibromotetradecane	7.7	n/d	−100
Isoeicosane	23.6	n/d	−100
Eicosane	63.0	3.0	−95.3
Pentacosane	38.9	1.9	−95.2
Hexacosane	40.0	7.5	−81.3
3,6-Dimethyldecane	30.4	6.4	−78.9
Hexatriacontane	19.6	22.1	+12.9
Hentriacontane	23.3	27.0	+15.8
Octacosane	30.5	27.0	−11.4
Heptacosane	7.3	28.0	+283.0
Triacontane	3.2	39.1	+1128.9
Dodecane	n/d	25.0	+100
Tetradecane	n/d	4.7	+100
**Alkane Total**	401.1	282.0	−29.7

**Table 3:** Distribution of sterols and lipid-derived aldehydes in *C. reinhardtii* under control and Cd-stressed conditions.

Compounds	Control (µg mg^−1^)	Cd-stressed (µg mg^−1^)	Relative change (%)
5α-Cholestan-3-one, 4,4-dimethyl, oxime	8.8	n/d	−100
Cycloeucalenyl acetate	4.6	n/d	−100
3-Deoxyestradiol	5.3	6.8	+28.5
Estradiol 3-tetrahydropyranyl ether	12.7	n/d	−100
Lanosterol	5.5	n/d	−100
Spongesterol	3.5	n/d	−100
Stigmasterol	2.8	n/d	−100
**Sterol Total**	43.2	13.3	−69.2
2-Nonenal, 2-pentyl (aldehyde)	n/d	49.6	+100

### 2.5 Statistical analysis

All experiments were performed with three independent biological replicates. Data are presented as mean ± standard deviation. Statistical analyses and graphical representations were performed using GraphPad Prism (version 9). Differences between control and Cd-treated groups were assessed using Student’s *t*-test, with statistical significance set at *p* < 0.05. Multivariate analysis, including principal component analysis, were performed using R software (version 2025.4.5.1).

## 3 Results

### 3.1 Cd toxicity assessment and EC_50_ determination

**Fig. 1:**
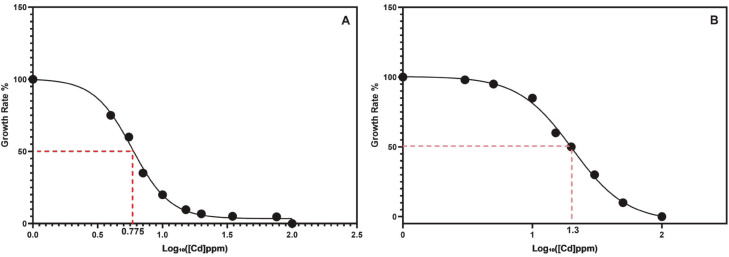
Dose-response analysis of *C. reinhardtii* exposed to Cd stress for 8 days. EC_50_ curve based on raw growth inhibition data showed a 50% growth reduction at 5.95 ppm (A), whereas normalized data indicated an EC_50_ value of 20 ppm (B).

Increasing Cd concentrations progressively inhibited *C. reinhardtii* growth, revealing a clear dose-dependent response (Fig. 1A). The initial dose-response assay performed in microplates yielded an EC_50_ value of 5.95 ppm after 8 days of exposure. To account for differences in stress sensitivity between culture systems, a cross-scale comparison was performed using a common reference concentration (100 ppm Cd) included in the initial dose-response assay. Under microplate conditions, this concentration resulted in complete growth inhibition (>100%), indicating an overestimation of stress intensity in small-volume systems. When tested in a larger, aerated culture (500 mL flask under continuous air bubbling), the same concentration produced approximately 100% inhibition at day 8, reflecting increased tolerance under improved physicochemical conditions. Based on this differential response, a normalization approach was applied to adjust the microplate-derived dose-response relationship to better represent the physiological behavior observed in aerated cultures. This adjustment resulted in a shift of the EC_50_ from 5.95 ppm to approximately 20 ppm. Accordingly, 20 ppm Cd was selected as the working concentration for subsequent experiments, as it represents a biologically relevant stress level under the culture conditions used for downstream physiological, biochemical, and lipidomic analyses.

### 3.2 Cd effects on *C. reinhardtii* growth and biomass productivity

**Fig. 2:**
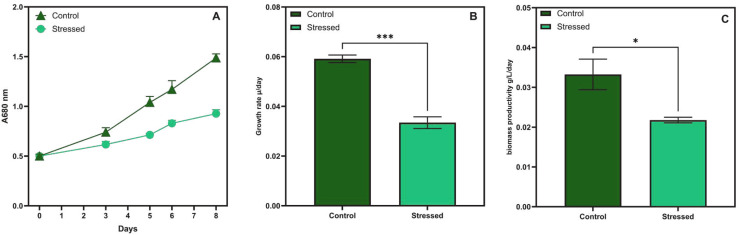
Growth suppression and productivity decline in *C. reinhardtii* under Cd stress. Time-course changes in OD_680nm_ values under control and Cd stress conditions (A). Specific growth rate (B). Biomass productivity (mg·L^−1^·d^−1^) (C).

As shown in Fig. 2A, Cd exposure significantly inhibited the growth of *C. reinhardtii*, as indicated by consistently lower OD_680nm_ values than those of the control throughout the cultivation period. Although OD_680nm_ increased over time under both conditions, control cultures maintained significantly higher values. By day 8, OD_680nm_ reached 1.49 ± 0.033 in control and 0.93 ± 0.02 in Cd-exposed cultures, corresponding to a 37.6% reduction relative to the control.

The specific growth rate (Fig. 2B) decreased from 0.059 ± 0.001 d^−1^ in the control to 0.033 ± 0.002 d^−1^ under Cd stress, corresponding to a 44% inhibition. Similarly, biomass productivity declined from 0.034 ± 0.003 *g*·L^−1^ d^−1^ to 0.021 ± 0.0012 *g*·L^−1^·d^−1^ Cd-exposed cultures (Fig. 2C), corresponding to a 38.2% reduction relative to the control.

### 3.3 Stress biomarkers in *C. reinhardtii* under Cd stress

**Fig. 3:**
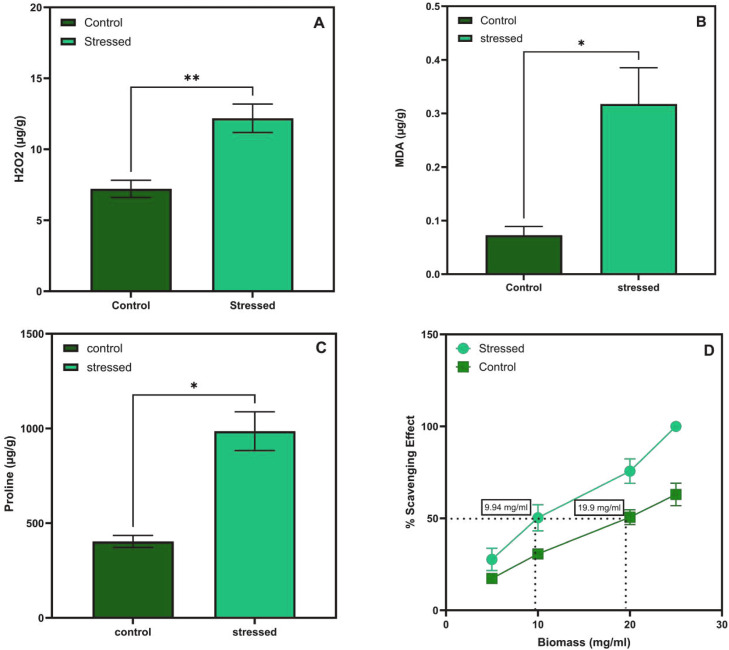
Oxidative stress biomarkers in *C. reinhardtii* under Cd(II) stress: H_2_O_2_ content (A), MDA levels (B), proline accumulation (C), and DPPH radical scavenging activity (D).

H_2_O_2_ content was significantly higher in Cd-exposed cultures than in the controls (Fig. 3A), reaching 12.18 ± 1.01 µg g^−1^ FW compared with 7.22 ± 0.60 µg g^−1^ FW, corresponding to an approximately 69% increase. Likewise, MDA content increased markedly (Fig. 3B), from 0.0728 ± 0.0163 µg g^−1^ FW in control to 0.3177 ± 0.0674 µg g^−1^ FW under Cd stress, representing an approximately 336.4% increase. Proline accumulation was also significantly enhanced (Fig. 3C), increasing from 403.7 ± 31.68 µg g^−1^ DW to 985.7 ± 102.55 µg g^−1^ DW corresponding to an approximately 144% increase. Radical scavenging activity was enhanced under Cd stress (Fig. 3D), as indicated by the lower IC_50_ value (9.95 ± 0.51 mg mL^−1^) compared with the control (19.9 ± 0.14 mg mL⁻^1^), corresponding to an approximately 50% reduction in IC_50_.

### 3.4 Cd-induced metabolic reprogramming in *C. reinhardtii*

**Fig. 4:**
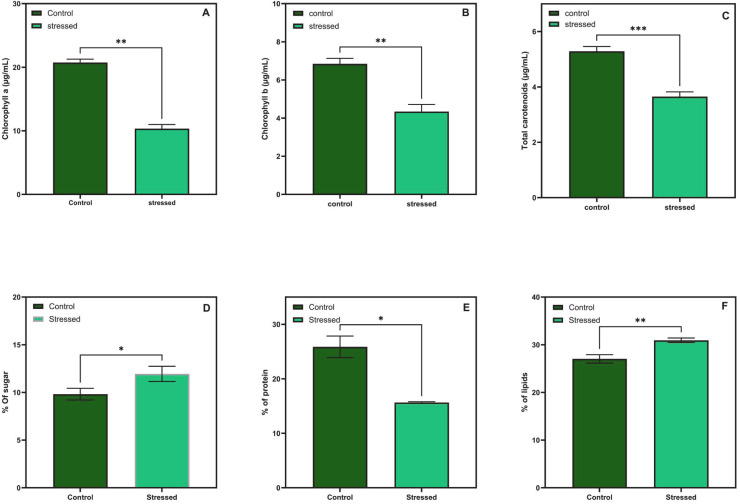
Cd-induced metabolic reprogramming in *C. reinhardtii*. Chlorophyll a content (A), chlorophyll *b* content (B), total carotenoids (C), total carbohydrate content (D), total protein content (E), and total lipid accumulation in control and Cd-stressed cultures (F).

Cd stress induced marked changes in primary metabolic parameters, including photosynthetic pigments, carbohydrates, proteins, and lipids (Fig. 4A–F). These metabolic alterations were accompanied by visible changes in culture appearance: control cultures retained an intense green coloration, whereas Cd-exposed cultures exhibited a pale yellow-green appearance by day 8 of cultivation. Spectral analysis of photosynthetic pigments further confirmed these observations; revealing altered absorbance profiles in Cd-treated samples compared with the controls (Fig. S_1_). Quantitative pigment analysis showed a significant decline under Cd stress. Chlorophyll *a* decreased by 50.2%, from 21.3 ± 0.4 to 10.6 ± 0.3 µg mL^−1^ (Fig. 4A), while chlorophyll *b* decreased by 33.3%, from 6.9 ± 0.2 to 4.6 ± 0.1 µg mL^−1^ (Fig. 4B). Total carotenoid content declined by 35.2%, from 5.4 ± 0.2 to 3.5 ± 0.1 µg mL^−1^ (Fig. 4C). In contrast, total carbohydrate content increased from 9.82 ± 0.61% in control to 11.95 ± 0.80% under Cd stress, corresponding to an increase of 21.7% (Fig. 4D). Protein content decreased from 25.88 ± 1.97% in controls to 15.65 ± 0.14% under Cd exposure, representing a reduction of 39.5% (Fig. 4E). Total lipid content increased from 27.05 ± 0.89% to 30.84 ± 0.61%, corresponding to an approximately 14% increase under Cd exposure (Fig. 4F).

### 3.5 GC-MS-based lipidomic remodeling in response to Cd stress

**Fig. 5:**
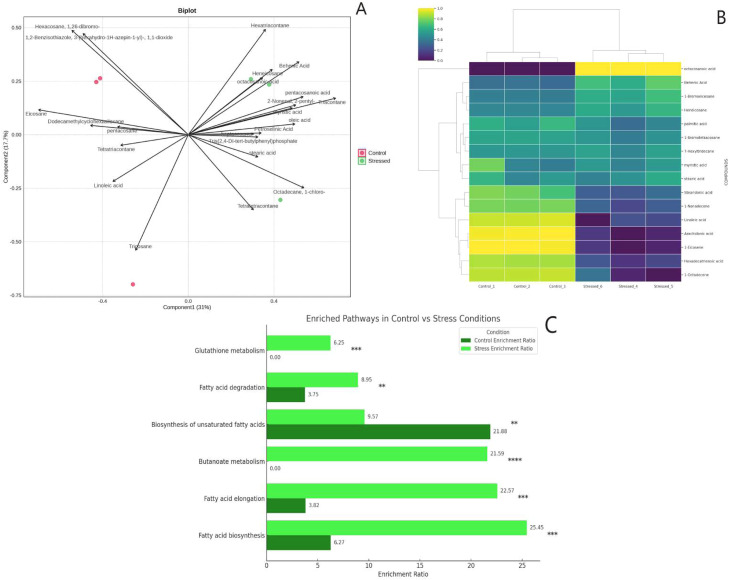
Lipidomic responses of *C. reinhardtii* under Cd stress. Biplot of lipidomic profiles in control and Cd-stressed cultures (A). Heatmap showing lipid accumulation under Cd exposure(B). Enrichment analysis of lipid-associated metabolic pathways under control and Cd stressed conditions (C).

A total of 95 lipid species were identified by GC-MS and included in the lipidomic analysis (Table S1). Principal component analysis and feature-impact biplot analysis revealed a clear separation between control and Cd-stressed samples (Fig. 5A). The first principal component (PC1) explained 31% of the total variance and clearly discriminated between the two experimental groups. This separation was primarily driven by changes in several lipid species, predominantly saturated. Among the most discriminant metabolites, 2-nonenal, 2-pentyl were more abundant under Cd stress. Pearson correlation analysis using this metabolite as reference feature revealed strong positive correlations with long-chain saturated fatty acids, including myristic, palmitic, behenic, and pentacosanoic acids, and strong negative correlations with polyunsaturated fatty acids (PUFAs), particularly linoleic and arachidonic acids (Fig. 5A). Hierarchical clustering further confirmed the distinct lipidomic profiles of control and Cd-stressed cultures (Fig. 5B). Cd exposure was associated with increased abundances of saturated fatty acids and alkanes, including octacosanoic acid, behenic acid and henicosane, together with decreased levels of unsaturated lipid species, such as linoleic acid, 1-octadecene, and arachidonic acid. A detailed classification of FAMEs, hydrocarbons, and sterols grouped by chemical class with relative abundances and percentage changes under Cd stress is presented in Table 1, Table 2, Table 3. KEGG pathway enrichment analysis further identified significant alterations in fatty acid biosynthesis, fatty acid elongation, butanoate metabolism, and glutathione metabolism (Fig. 5C).

## 4 Discussion

Cd exposure markedly inhibited the growth of *C. reinhardtii*, confirming its pronounced toxicity toward freshwater microalgae. Reported EC_50_ values for Cd vary considerably depending on culture medium, metal speciation, exposure duration, inoculum density and cultivation system [27,28]. In the present study, the absence of organic carbon and chelating agents such as EDTA in BG_11_ medium likely increased Cd bioavailability and enhanced its inhibitory effect, contributing to lower EC_50_ values [12,29]. Moreover, culture scale further modulated stress intensity: microplate assays yielded a lower EC_50_ (5.95 ppm), indicating heightened sensitivity, while cross-scale validation using 100 ppm Cd confirmed an overestimation relative to aerated 500 mL flask cultures. Applying a normalization approach shifted the effective EC_50_ to 20 ppm, which was selected as a biologically relevant sublethal concentration for subsequent analyses. Under these conditions, Cd significantly reduced specific growth rate and biomass productivity, indicating impaired cellular performance [30,31]. These observations are consistent with previous reports showing that Cd disrupts photosynthesis, nutrient assimilation, and carbon fixation through inhibition of enzymatic activities, displacement of essential metal cofactors, and structural damage to chloroplasts [12,32,33]. The resulting reduction in growth likely reflects a shift in cellular resource allocation from biomass production toward stress adaptation and maintenance of cellular homeostasis, as previously described for *Chlamydomonas* species exposed to Cd [34] and Pb [35].

Although Cd is not a redox-active metal, it disrupts cellular redox homeostasis by impairing electron transport in chloroplasts and mitochondria, leading to excessive ROS production and oxidative stress as evidenced by the significant accumulation of H_2_O_2_ [36]. The resulting lipid peroxidation, reflected by elevated MDA levels, compromises membrane integrity by altering membrane fluidity, permeability, and the function of membrane-associated proteins [11,37]. In parallel, the significant accumulation of proline and the enhanced antioxidant capacity indicate activation of non-enzymatic defense systems, with proline acting as an osmoprotectant, molecular chaperone, and redox buffer to reinforce cellular tolerance under Cd stress [38,39].

Pigment analysis revealed a clear chlorosis phenotype by day 8, with significant reductions in chlorophyll a, chlorophyll *b*, and total carotenoids (Fig. 4), indicating severe disruption of the photosynthetic apparatus. Cd inhibits key enzymes in chlorophyll biosynthesis and activates chlorophyll degradation *via* chlorophyllase and ROS-mediated porphyrin cleavage [40,41]. Carotenoid depletion is particularly detrimental, as these molecules are essential photoprotective antioxidants; their loss exacerbates oxidative damage and pigment degradation in a positive feedback loop [42]. Biochemical profiling further revealed reduced protein content accompanied by increased carbohydrates and total lipid accumulation (Fig. 4), indicating a shift in carbon allocation from protein biosynthesis toward stress-adaptive metabolism. Carbohydrates likely serve as osmolytes and energy reserves, while lipid accumulation, including enhanced triacylglycerols biosynthesis, contributes to membrane stabilization and protection against oxidative damage [3,43,44].

To further elucidate the metabolic basis of this reprogramming, GC-MS-based lipidomic profiling revealed extensive remodeling of lipid composition under Cd stress (Table 1). The most prominent change was a marked depletion of PUFAs, including C_16:3_ isomers, C_18:4_, and arachidonic acid (C_20:4_), accompanied by an almost twofold increase in saturated fatty acids (SFAs; 516.1 µg mg^−1^ to 1037.4 µg mg^−1^) and the appearance of several monounsaturated fatty acids (MUFAs; C_16:1_, C_18:1_ isomers, C_19:1_). This shift from unsaturated to more saturated lipids is consistent with preferential ROS-mediated peroxidation of PUFAs and likely represents an adaptive mechanism to reduce membrane susceptibility to oxidative damage [45,46]. Because thylakoid membranes are enriched in PUFAs that are essential for maintaining photosystem organization and membrane fluidity, their depletion may contribute to the observed decline in photosynthetic pigments by destabilizing chlorophyll-protein complexes within photosystem II and the light-harvesting complex, thereby promoting chlorophyll and carotenoid degradation [47,48]. Alkane and sterol profiles also underwent substantial remodeling under Cd stress (Table 2, Table 3). Branched and halogenated alkanes detected in control cells were largely replaced by shorter- and long-chain linear alkanes including hentriacontane and triacontane, suggesting reorganization of surface lipid composition that may contribute to enhanced membrane stability [53].

Total sterol abundance decreased markedly, from 43.2 µg mg^−1^ to 13.3 µg mg^−1^ (approximately 69%), with the complete loss of lanosterol, stigmasterol, and cycloeucalenyl acetate. Together with the accumulation of SFAs, sterol depletion is consistent with membrane remodeling that may enhance membrane stability and reduce Cd permeability under stress. Notably, 2-nonenal was detected exclusively in Cd-treated cells (Table 3). This α,β-unsaturated aldehyde is a well-established product of linoleic and linolenic acid peroxidation precisely the PUFA species depleted under stress providing direct in vivo evidence of active lipid peroxidation and directly linking the lipidomic signature to the elevated MDA levels measured biochemically [49,50]. Its strong positive correlation with long-chain SFAs (Fig. 5B) positions 2-nonenal as a key biomarker integrating oxidative lipid damage with adaptive metabolic reprogramming.

**Fig. 6:**
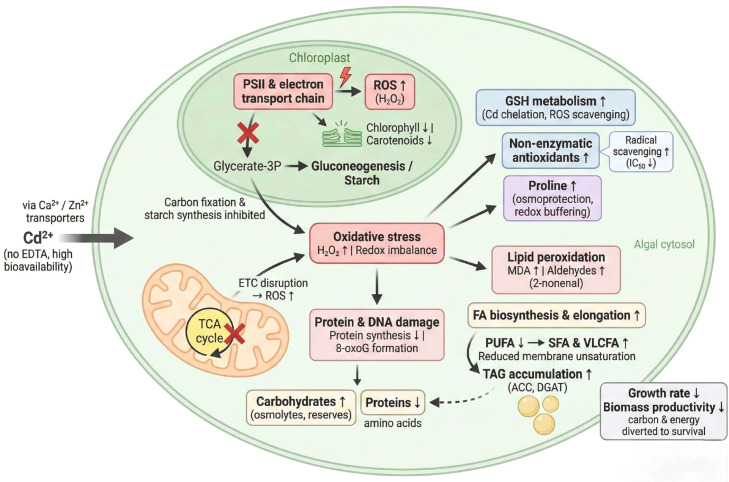
Coordinated metabolic, biochemical, and physiological responses of *C. reinhardtii* to Cd stress: Cd uptake through Ca^2^⁺/Zn^2^⁺ transporters affects photosynthetic and mitochondrial electron transport, increasing ROS production and leading to redox imbalance. Oxidative stress promotes lipid peroxidation and activation of antioxidant defense systems, accompanied by lipid metabolic reorganization, including decreased PUFA levels, increased saturated and very long-chain fatty acids, and enhanced TAG synthesis. Cd stress also induces carbon metabolic reprogramming, shifting carbon allocation from protein toward carbohydrates and lipids, ultimately reducing growth rate and biomass production. Solid lines represent metabolic fluxes, dashed lines indicate inferred flux relocations, and red crosses indicate reduced or inhibited processes.

Pathway enrichment analysis identified significant alterations in fatty acid biosynthesis, fatty acid elongation, butanoate metabolism, and glutathione metabolism. Enrichment of biosynthesis and elongation pathways explains the accumulation of very-long-chain SFAs (C_22:0_, C_25:0_, C_28:0_), which modulate membrane physical properties and stabilize membrane-associated proteins [51,52]. Activation of glutathione metabolism highlights its central role in Cd detoxification through HMs chelation and ROS scavenging, while butanoate metabolism may provide alternative carbon fluxes and NADPH regeneration under conditions of photosynthetic and mitochondrial dysfunction [53]. Concomitant activation of fatty acid degradation contributes to ROS generation *via* β-oxidation under Cd-induced dysfunction [54], accounting for the marked H_2_O_2_ elevation observed. This coupling between lipid turnover and redox imbalance is conceptually depicted in Fig. 6, where mitochondrial and chloroplastic perturbations converge on a central oxidative stress node. Proline accumulation further integrates into this framework, as intermediates from central carbon metabolism feed glutamate-derived proline biosynthesis [55], reinforcing cellular tolerance through osmoprotection and redox buffering. Collectively, these processes constitute a coordinated survival strategy encompassing membrane remodeling, antioxidant activation, and carbon reallocation that underpins reduced biomass productivity as *C. reinhardtii* transitions from growth-oriented to survival-oriented metabolism under Cd stress [56,57].

## 5 Conclusion

This study provides an integrated characterization of the adaptive responses of *C. reinhardtii* to Cd stress by combining physiological, biochemical, GC-MS-based lipidomic, and pathway enrichment analyses. Cd exposure induced pronounced oxidative stress, impaired growth and photosynthetic performance, and triggered extensive metabolic reprogramming characterized by carbohydrate and lipid accumulation, protein depletion, and activation of antioxidant defenses. Lipidomic profiling revealed a marked shift from polyunsaturated to saturated fatty acids, accompanied by remodeling of hydrocarbon and sterol composition, highlighting membrane lipid reorganization as a central adaptive response to Cd-induced oxidative stress. KEGG pathway enrichment further identified fatty acid biosynthesis, fatty acid elongation, glutathione metabolism, and butanoate metabolism as major pathways involved in this metabolic adaptation. Beyond improving our understanding of microalgal responses to Cd toxicity, these findings demonstrate how controlled metal stress redirects carbon metabolism toward stress-associated lipid remodeling. This knowledge provides a basis for optimizing microalgal cultivation strategies that integrate wastewater phycoremediation with the production of lipid-rich biomass for biotechnological applications. Future studies integrating transcriptomics and proteomics with lipidomics will further clarify the regulatory networks underlying these adaptive responses and support the development of stress-resilient microalgal strains for sustainable phycoremediation and biomass valorization.

## Funding

This research was supported by a PhD studentship funded by Mohammed VI Polytechnic University.

## CRediT authorship contribution statement

**Mohammad Aadil:** Writing – original draft, Visualization, Validation, Methodology, Investigation, Formal analysis, Data curation. **Nihad Sahri:** Writing – review & editing, Validation, Formal analysis, Data curation, Conceptualization. **Mutale Joan Chanda:** Visualization, Validation, Methodology. **Soufiane Fal:** Methodology, Investigation, Data curation. **Rachid Benhida:** Writing – review & editing, Validation, Supervision, Resources, Project administration, Funding acquisition. **Hicham El Arroussi:** Writing – review & editing, Validation, Supervision, Conceptualization. **Mohammed Danouche:** Writing – review & editing, Writing – original draft, Validation, Supervision, Project administration, Methodology, Investigation, Conceptualization.

## Declaration of competing interest

The authors declare no competing interests.

## Data Availability

No data was used for the research described in the article.

## Appendix Supplementary materials

Image, application 1
