# Supplementary material for: Adaptive responses of Chlamydomonas reinhardtii to cadmium stress: physiological, biochemical, and lipidomic insights
Source: Biotechnol Rep (Amst). 2026 Jul 8;51:e00970. doi: 10.1016/j.btre.2026.e00970 (PMC13400856; doi:10.1016/j.btre.2026.e00970)

**Supplementary material**

**Table S1.** Identified metabolites and their relative abundances in control and Cd-stressed *C. reinhardti.*

| Compounds | Control (µg mg⁻¹) | Control  (µg mg⁻¹) |
| --- | --- | --- |
| 1,14-Dibromotetradecane | 7.656 | 0 |
| 1,2-Benzisothiazole, 3-(hexahydro-1H-azepin-1-yl)-, 1,1-dioxide | 44.137 | 0 |
| 16-methylheptadecanoic acid | 88.822 | 0 |
| 17,21-Dimethylheptatriacontane | 8.944 | 0 |
| 1-Bromo-4-bromomethyldecane | 1.264 | 0 |
| 1-Chlorodocosane | 2.122 | 0 |
| 1-Eicosene | 7.687 | 0 |
| 1-Hexadecanesulfonic acid, 3,5-dichloro-2,6-dimethyl-4-pyridyl ester | 2.411 | 0 |
| 1-Nonadecene | 1.831 | 0 |
| 2,5-Furandione, 3-dodecyl- | 2.034 | 0 |
| 2,6,10-Trimethyltridecane | 1.681 | 0 |
| 2-Methylhentriacontane | 13.976 | 0 |
| 3-Methylheneicosane | 2.985 | 0 |
| 5alpha-Cholestan-3-one, 4,4-dimethyl-, oxime | 8.808 | 0 |
| Arachidonic acid | 13.332 | 0 |
| ascorbyl palmitate | 4.231 | 0 |
| Chloroform | 1.696 | 0 |
| Chloroicosane | 2.038 | 0 |
| Cycloeucalenyl acetate | 4.555 | 0 |
| Cycloheptasiloxane, tetradecamethy | 1.368 | 0 |
| Cycloheptasiloxane, tetradecamethyl-l- | 1.399 | 0 |
| Cyclomethicone 5 | 35.954 | 0 |
| Dimethyl terephthalate | 4.295 | 0 |
| Dodecamethylcyclohexasiloxane | 10.452 | 0 |
| Dodecyl ether | 12.592 | 0 |
| Dotriacontane, 1,32-dibromo- | 5.971 | 0 |
| Estradiol 3-tetrahydropyranyl ether | 12.717 | 0 |
| Hexacosane, 1,26-dibromo- | 4.785 | 0 |
| Hexadecatrienoic acid | 3.599 | 0 |
| Isoeicosane | 23.561 | 0 |
| ISOPALMITIC ACID | 8.094 | 0 |
| lanosterol | 5.541 | 0 |
| Linoleic acid | 16.547 | 0 |
| n-Tetradecyltrichlorosilane | 3.153 | 0 |
| Octacosane, 2-methyl- | 4.928 | 0 |
| PropionicÂ acid | 2.171 | 0 |
| Pyridine-3-carboxamide, oxime, N-(2-trifluoromethylphenyl) | 96.479 | 0 |
| Spongesterol | 3.484 | 0 |
| Stearidonic acid | 2.245 | 0 |
| stigmasterol | 2.811 | 0 |
| tetracosane | 0.553 | 0 |
| Tricosane | 15.835 | 0 |
| Pentatriacontane | 0 | 0.154 |
| Heneicosane, 11-pentyl- | 0 | 0.322 |
| Docosane | 10.027 | 0.395 |
| Methyl 12-methyltridecanoate | 1.461 | 0.638 |
| Tetracontane | 0 | 0.685 |
| 2-Octadecoxyethanol | 21.738 | 0.761 |
| 1-Hexacosene | 0 | 0.858 |
| 1-Bromodocosane | 2.207 | 0.981 |
| nonadecane | 0 | 1.327 |
| Octadecane, 1,1'-[(1-methyl-1,2-et | 0 | 1.552 |
| Octadecane, 1-(ethenyloxy)- | 0 | 1.644 |
| pentacosane | 38.876 | 1.878 |
| 2-Methyltriacontane | 5.416 | 1.987 |
| 1-Bromotetracosane | 0 | 2.311 |
| 8-methyldecanoic acid | 0 | 2.368 |
| Disulfide, di-tert-dodecyl | 4.443 | 2.491 |
| Ethyl undecanoate | 1.958 | 2.541 |
| oleic acid | 0 | 2.584 |
| cis-10-Nonadecenoic acid | 0 | 2.841 |
| Octadecane, 1-chloro- | 0 | 2.885 |
| Tritetracontane | 0.249 | 2.954 |
| Eicosane | 62.998 | 2.962 |
| Docosane, 1,22-dibromo- | 0.944 | 3.403 |
| 7-Hexyltridecane | 0 | 3.797 |
| tetradecane | 0 | 4.695 |
| 3-Methyltritriacontane | 0 | 4.996 |
| Octadecane, 1-iodo- | 15.623 | 5.465 |
| 1-Octadecene | 8.702 | 5.645 |
| 1-Docosene | 0 | 5.761 |
| palmitoleic acid | 0 | 5.894 |
| 3,6-Dimethyldecane | 30.431 | 6.413 |
| Heneicosane | 0 | 6.566 |
| 3-Deoxyestradiol | 5.267 | 6.767 |
| elaidic acid | 0 | 7.075 |
| Petroselinic Acid | 0 | 7.298 |
| hexacosane | 39.951 | 7.509 |
| 3,8-Dimethyldecane | 0 | 7.698 |
| Tetratriacontane | 11.5141 | 7.818 |
| 1-Bromoeicosane | 0 | 8.558 |
| Tetratetracontane | 2.746 | 9.334 |
| Heneicosane, 11-decyl- | 0 | 10.041 |
| Hexadecane, 1-iodo- | 0 | 13.727 |
| OCTADECYLTRICHLOROSILANE | 19.572 | 17.623 |
| myristic acid | 0 | 21.522 |
| Hexatriacontane | 19.554 | 22.057 |
| Dodecane | 0 | 24.983 |
| Behenic Acid | 2.985 | 26.519 |
| hentriacontane | 23.322 | 26.994 |
| octacosane | 30.491 | 27.029 |
| heptacosane | 7.331 | 28.011 |
| Triacontane | 3.155 | 39.099 |
| Tris(2,4-DI-tert-butylphenyl)phosphate | 0 | 45.425 |
| 2-Nonenal, 2-pentyl- | 0 | 49.641 |
| pentacosanoic acid | 0 | 69.333 |
| octacosanoic acid | 0 | 89.323 |
| stearic acid | 103.406 | 251.649 |
| palmitic acid | 312.767 | 579.039 |

**Figure S1.** Distribution of metabolite abundance values before and after normalization.


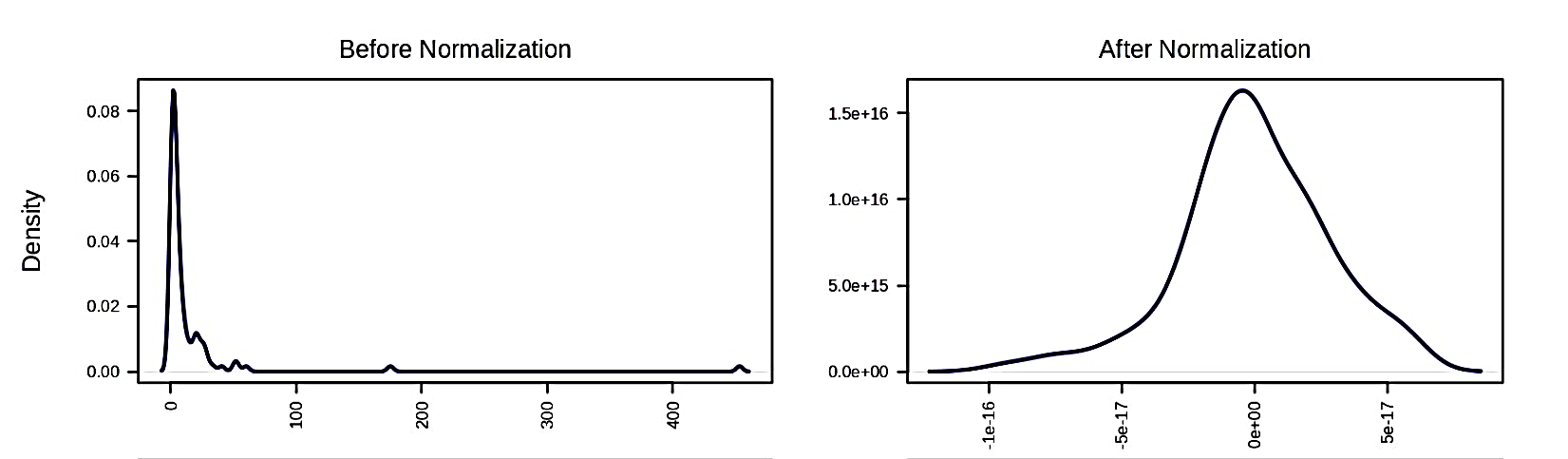


**Figure S2.** Comparison of metabolite abundance ranges before and after normalization.


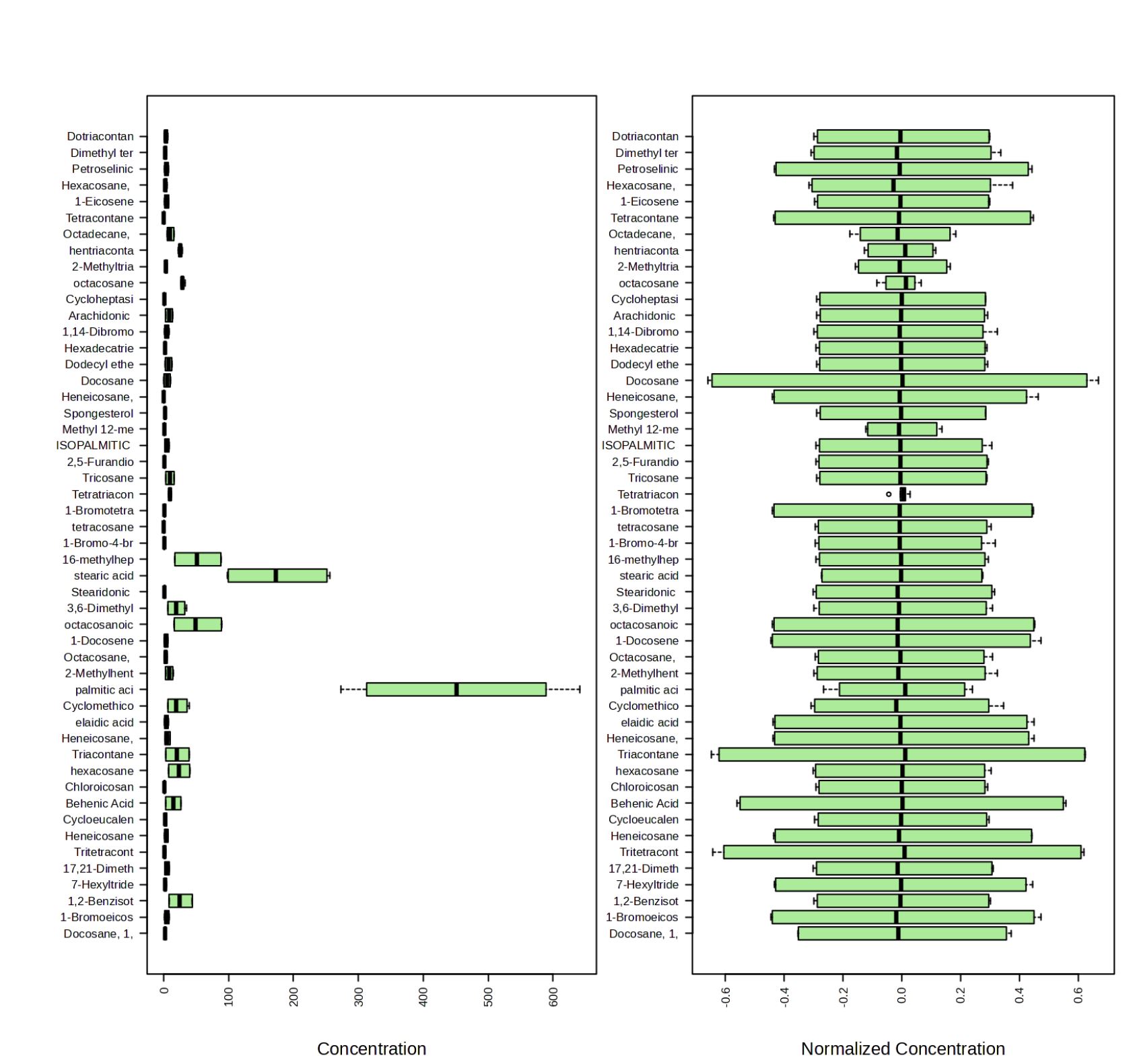

Supplement: Supplementary file 1 [file mmc1.docx]
